# Location‐specific pathology analysis of the monopodial pulmonary vasculature in a rabbit model of bronchopulmonary dysplasia—A pilot study

**DOI:** 10.14814/phy2.15747

**Published:** 2023-06-26

**Authors:** Jonas Labode, David Haberthür, Ruslan Hlushchuk, Yannick Regin, Andre George Gie, Thomas Salaets, Jaan Toelen, Christian Mühlfeld

**Affiliations:** ^1^ Hannover Medical School Institute of Functional and Applied Anatomy Hannover Germany; ^2^ Biomedical Research in Endstage and Obstructive Lung Disease Hannover (BREATH), Member of the German Center for Lung Research (DZL) Hannover Germany; ^3^ Institute of Anatomy University of Bern Bern Switzerland; ^4^ Department of Development and Regeneration KU Leuven Leuven Belgium; ^5^ Department of Paediatrics and Child Health, Faculty of Medicine and Health Sciences Stellenbosch University Cape Town South Africa

**Keywords:** branching analysis, cluster analysis, light microscopy, microcomputed tomography, monopodial lung, pulmonary vasculature

## Abstract

The mammalian pulmonary vasculature consists of functionally and morphologically heterogeneous compartments. When comparing sets of lungs, for example, in disease models or therapeutic interventions, local changes may be masked by the overall heterogeneity of the organ structure. Therefore, alterations taking place only in a sub‐compartment may not be detectable by global analysis. In the monopodial lung, the characterization of distinct vessel groups is difficult, due to the asymmetrical branching pattern. In this pilot study, a previously established method to classify segments of the monopodial pulmonary arterial tree into homogeneous groups was employed. To test its suitability for experimental settings, the method was applied to a hyperoxia (HYX, ≥95% oxygen) rabbit model of bronchopulmonary dysplasia and a normoxic control group (NOX, 21% oxygen). The method allowed the identification of morphological differences between the HYX and the NOX groups. Globally visible differences in lumen diameter were pinpointed to specific lung regions. Furthermore, local changes of wall dimension and cell layers in single compartments, that would not have been identifiable in an unfocused analysis of the whole dataset, were found. In conclusion, the described method achieves a higher precision in morphological studies of lung disease models, compared to a common, global analysis approach.

## 1 INTRODUCTION

The morphological characteristics of airway and vessel walls in the mammalian lung change significantly along their way from the entry at the hilus to their final branching into alveoli and septal capillaries, respectively (Townsley, 2011).

Due to the heterogeneity of the morphological characteristics of airway and vessel branches (Huang et al., 1996; Plopper et al., 1983), it is likely that different compartments within the branching tree behave differently under pathological conditions. Thus, when comparing lungs of different experimental groups, it would be more informative to analyze airway and vessel compartments of equal location within the airway and vessel tree, instead of comparisons of global (whole lung) measurements, which might mask local changes due to a dilution effect.

The practical application of this principle is faced with various challenges. In histology, analyses are often performed on thin microscopic sections, where spatial context is limited. It is thus not obvious where a vessel cross section which is visible in a slice was located along the vessel tree in the original organ structure.

Without additional context, vessel profiles can only be grouped coarsely according to their size/diameter or their wall thickness. These parameters may also change due to the underlying pathological process, thus leading to even less precise groupings. For this reason, Grothausmann et al. (2021) demonstrated a process to insert color‐coded generation markings into the vessel cross sections visible in microscopic images and thereby provide the missing spatial context for site‐specific analyses.

A second challenge is the selection of a metric for the grouping of vessels into morphological and functional homogeneous groups. The relatively symmetrical branching of the human lung allows a grouping into generations (see Ochs & Weibel, 2008), which are morphologically and functionally similar. This is performed by counting the bifurcations between the root of the vessel tree and the location of a specific vessel. The situation is different in other mammalian lungs, as the vessels of non‐primate lungs branch in an asymmetrical pattern. The degrees of asymmetry differ between species. Common laboratory animals such as rat or rabbit exhibit a highly asymmetric monopodial branching, characterized by one long central airway with smaller lateral branches (Hyde et al., 2006; Phalen & Oldham, 1983). Here, generations are insufficient for classification. The literature lists multiple competing classification algorithms with the goal of dividing the monopodial vessel tree into homogeneous subgroups, for example, Strahler orders (Strahler, 1957), Horsfield orders (Horsfield et al., 1982) or fractal generations (Wang & Kraman, 2004). To address this issue, Labode et al. (2022) established a workflow to measure the performance of different grouping schemes.

The combination of the methods of Grothausmann et al. (2021) and Labode et al. (2022) promises to enable the collection of location‐specific measurements in the monopodial lung and thus allow comparisons between compartments of equal or at least similar biological functionality.

The aim of the study presented here was to employ this combined workflow to a real‐world case, by using it to detect pathological changes of the pulmonary arterial structure between control and experimental rabbit lungs in a model of bronchopulmonary dysplasia (BPD).

BPD is a condition that affects prematurely born infants with insufficiently developed lungs (Northway et al., 1967). The currently relevant form of BPD affects mainly very immaturely born babies (between gestation weeks 24 and 28) and is characterized by arrested lung development with impaired alveolar and vascular development being the pathological hallmarks of the human disease (Abman, 2001; Solaligue et al., 2017). This disruption of development can further lead to an increased pulmonary vascular resistance, causing eventual pulmonary hypertension and right heart strain (Malloy & Austin, 2021).

It was expected, that the heterogeneous pathological changes along the vascular system, such as narrowing of vessel diameters or abnormal smooth muscle proliferation into small peripheral arteries (Mourani & Abman, 2015) would present an ideal test case for the developed method. The rabbit model of BPD has proved to replicate the lung development changes found in human BPD (Jiménez et al., 2016; Richter et al., 2014) and is thus a well‐established method for the study of symptoms and intervention strategies in BPD (Gie, Regin, et al., 2020; Gie, Salaets, et al., 2020). It is therefore used in this study. An extensive design‐based stereological analysis of the implementation of the rabbit BPD model, as used in the present study, is detailed in Mühlfeld et al. (2021).

The workflow detailed here is an extended version of that applied in Labode et al. (2022). It consists of the preparation of the lung sample material, followed by both non‐destructive and destructive image acquisition. On this material, morphological data acquisition was conducted. The best performing grouping algorithm for the sample material was identified. It was then used to attribute collected, morphological measurements of the lung vasculature to distinct groups, homogeneous in function and morphology. On this basis, a location‐specific statistical comparison of test and control samples was executed. It was then evaluated whether this approach was practical and if the location‐specific analysis was able to provide more detailed results compared to an unspecific analysis of the collected measurements.

The sample size of this pilot study was limited to two animals per group. We therefore did not expect to generate an adequate and representative profile of the pathological changes caused by BPD in the arterial vasculature. Instead, this pilot study will serve as the first comparative analysis approach based on the methods described above on actual study data. This will allow a review of the analysis process and its suitability for the intended purpose.

## 2 METHODS

### 2.1 Rabbit lung processing

The study was approved by the Ethics Committee for Animal Experimentation of KU Leuven, Belgium (P081/2017). Four rabbit pups were delivered by cesarean section at 28 days of pregnancy (term = 31 days). The sex of the rabbit pups was not determined or controlled for. They were transferred to a custom humidity‐ and temperature‐controlled incubator (Okolab, Pozzuoli, 121. Italy) for 7 days. Environmental conditions were set to 32°C and 50% humidity. Two of the animals were exposed to hyperoxia (≥95% oxygen) while the other two were kept in normoxic conditions. Table 1 lists the weight of the animals at time of birth and at the time of euthanization. The right lung volumes were determined in a parallel study and are listed for comparison purposes. Animals kept in normoxic conditions are denoted NOX, animals exposed to hyperoxia are marked HYX.

**TABLE 1:** Animal data.

	Body weight (g) at birth	Body weight (g) at day 7	Right lung volume (cm^3^)
NOX1	40.7	56.5	1.01
NOX2	38.4	49.5	1.76
HYX1	44.7	50.9	1.08
HYX2	42.3	54.3	1.67

On day 7, the rabbits were anesthetized using 35 mg/kg of ketamine and 6 mg/kg of xylazin. Perfusion fixation was carried out as described previously (Grothausmann et al., 2021). In short, after cannulation of the trachea, a recruitment maneuver was performed, and a positive end expiratory pressure of 5 cm H_2_O was maintained. The right ventricle was catheterized, and the lungs were perfused with an aldehyde fixative at a pressure of 25 cm H_2_O. The animals were euthanized during the perfusion process by exsanguination. The heart–lung block was excised and stored in the same fixative at 4°C until further processing.

The left lung was separated from the heart–lung block and embedded in toto in glycol methacrylate (Technovit 7100; Heraeus Kulzer). The right lungs were removed for a separate study. Briefly, the lungs were postfixed with 1% osmium tetroxide and 1% uranyl acetate, subsequently dehydrated in an ascending acetone series, and finally embedded in glycol methacrylate. According to the stereological principles outlined in Baddeley et al. (1986), the lungs were rotated around a vertical axis through apex and base of the lung before embedding.

### 2.2 Microcomputed tomography imaging

After the extraction and embedding of the lungs, microcomputed tomography imaging (μCT) was performed to generate non‐destructive 3D images of the organ structure. The imaging device was a SkyScan 1272 high‐resolution microtomograph (Control software version 1.1.19; Bruker microCT). This machine is equipped with a Hamamatsu L11871_20 X‐ray source and a XIMEA xiRAY16 camera. A tube voltage of 80 kV and a tube current of 125 μA were used. Additionally, an aluminum filter (1 mm) was put in place to filter the X‐ray spectrum prior to incidence onto the sample. The large size of the samples made it necessary to record two overlapping scans per sample and stitch them laterally. Each scan consisted of 488 projections with a resolution of 3104 × 1091 pixels at every 0.4° over a 180° sample rotation.

Exposure time was set to 2247 ms for each projection; five projections were averaged to one for an image noise reduction. This setup resulted in a total scan time of approximately 8 h per sample. The software NRecon (Version 1.7.4.2; Bruker microCT) was used for the reconstruction of the projection images into a 3D stack of images. A ring artifact correction of 7 was employed. The whole process resulted in datasets with an isometric voxel size of 7.0 μm.

### 2.3 Lung sectioning

As the lung tissue was taken from a study incorporating a stereological assessment, the sampling procedure contained appropriate randomization steps (see Ochs & Schipke, 2021). The lungs were exhaustively sectioned parallel to the vertical axis on a microtome for light microscopic (LM) imaging. Two different sampling schemes were employed. Several series of 24 slices each were generated. As there was no previous experience with image registration of that kind, it was unknown which slice thickness would be appropriate. Therefore, for the lungs NOX1 and HYX1, sample collection was performed every 300 μm, with a slice thickness of 4 μm. For the lungs NOX2 and HYX2, the space between sample collections was set to 100 μm with a section thickness of 2 μm.

Starting with a random number between 1 and the step size of either 100 or 300 μm, 24 consecutive sections of 2 μm (resp. 4 μm) thickness were collected and mounted on glass slides. The next 50 (resp. 75) sections (100 or 300 μm of tissue) were discarded, and the following 24 sections were again mounted on glass slides. This procedure was repeated until the whole lung was sectioned. The selected sections were then stained with toluidine blue and digitized using a Zeiss Axioscan (Zeiss) slide scanner.

### 2.4 Segmentation and classification

**FIGURE 1:**
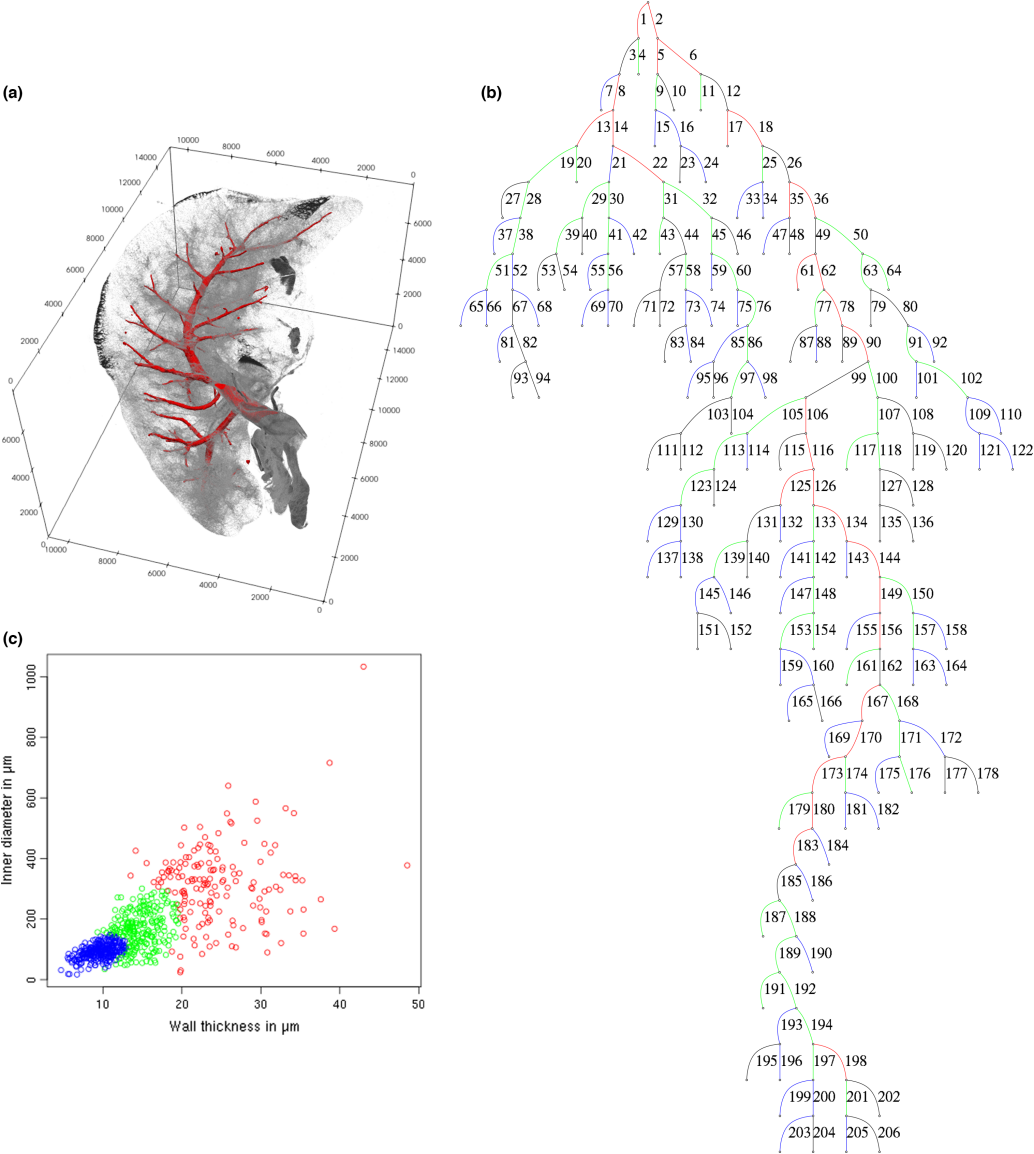
The workflow starts at the segmentation of the vessel tree from the μCT image, here the segmentation of the pulmonary artery in the left rabbit lung NOX2 is displayed (a). The branching structure is then extracted from the segmentation image (b). The identified vessel segments are assigned an unique identifier number and are then clustered according to their morphological features (c). Here the GMM is used as clustering method. Clusters are determined by identifying Gaussian distribution patterns in the dataset. Scale (a) in μm. Color in (b) according to (c), black = no measurement available.

The μCT volume images were digitally segmented to extract an image of the arterial tree (Figure 1a). As described in Labode et al. (2022), the volume image of the artery was then reduced to a graph (see Figure 1b).

Each edge in the graph (section between two bifurcations or a bifurcation and an end point) was assigned a unique ID. This attribution served as the basis for location‐specific measurements. Additionally, these graph segments formed the elements that were grouped into homogeneous vessel classes (Figure 1c), to allow comparison of morphologically similar sub regions of the vessel tree (see Section 2.7).

The μCT images with a resolution of 7 μm allowed a segmentation of the pulmonary artery down to vessels with a minimal lumen diameter of about 17 μm. Due to a large amount of noise in the images, the smaller, more distal artery segments could not be segmented reliably and are thus underrepresented in the following analysis.

In addition to this global, imaging‐based phenomenon, there were local deviations caused by the tissue preparation process. In the lung HYX1, the arterial root connecting the vessels supplying the two lobes of the lung could not be properly segmented, as it had been damaged in the extraction process. This resulted in two separate vessel trees for this sample. To allow for a connected, graph‐based grouping and correct graph analysis, an artificial node was thus manually introduced to connect these two trees. As a consequence, measurements could not be collected for this area. Additionally, in the sample NOX1, a distal section of the central artery in the upper lobe collapsed during perfusion and could thus not be segmented or further analyzed. This caused one vessel segment with a comparatively large diameter to be introduced into order 1 of the order and Strahler order algorithms (described below).

### 2.5 Combination μCT and LM

The ID marked volume images of the arterial trees were overlaid on the high‐resolution LM volume sections, using the workflow developed by Grothausmann et al. (2021).

This process consisted of registering the LM slices of each volume section to each other, to form a volume image with enough distinct features for further registration. In the next step, the μCT volume image was registered to each of these LM volumes. The registration of the μCT image to the LM image was chosen over the opposite approach, as not to distort the high‐resolution LM images and thus reduce image quality. The registration parameters resulting from this process were subsequently employed to the segmentation image of the arterial tree. As a result, the segmentation image is congruent with the LM images and can be overlaid on them, providing color markings for 3D context in the 2D high‐resolution LM images.

The differences in slice thickness of the tissue sections did not affect the registration process in any significant way. Both sampling protocols delivered suitable sets of LM images.

### 2.6 Morphometric data acquisition

The feature measurements were performed in the color‐coded LM images. Of each volume section (usually 24 images, less if slices were damaged in the cutting process and were thus unfit for registration), the first, middle, and last slice were analyzed.

**FIGURE 2:**
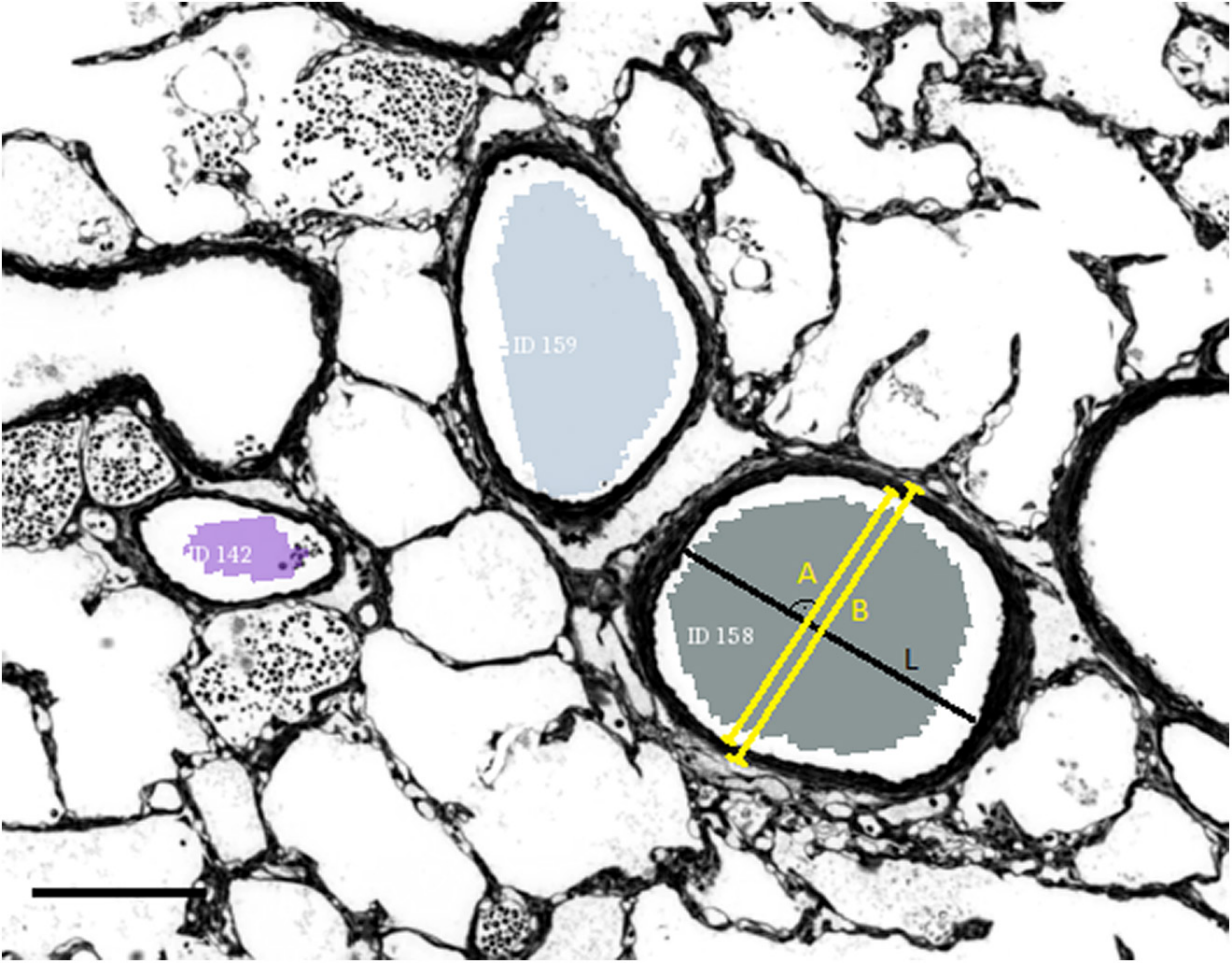
Measuring process demonstrated on the vessel with the ID 158. Measurements are taken perpendicular to the long axis (L) of the vessel cross section. Measured were lumen diameter (A) and vessel diameter including the tunica media (B). The wall thickness was calculated by subtracting A from B and dividing the result by two. Scale bar: 300 μm.

The lumen diameter and wall thickness were measured at the largest diameter perpendicular to the long axis of every visible vessel cross section in each image. For this task, Fijis (Schindelin et al., 2012) line drawing tool was employed. That is, a line was drawn once across the lumen and measured to acquire the diameter. For the determination of the wall thickness, a second line was then drawn from one media border over media, intima, lumen, and intima and media on the opposing side. From this measurement, the lumen was subtracted, and the result divided by two. An adventitia was rarely observed and thus excluded from the analysis. Figure 2 demonstrates the measuring process.

Additionally, smooth muscle cell layers in the arterial walls were counted. Counting was performed on 40× LM images in their original toluidine blue staining, opposed to the monochromatic images resulting from the digital registration process seen in Figure 2.

The measurements were collected for each ID marked in each combination image. If more than one measurement was recorded for the same ID (across multiple images), an average was calculated.

Table 2 lists the volume section numbers as well as the number of identified and measured vessel segments and muscle cell layer counts per lung. Layer counts were performed on less vessel segments than measurements were taken for, as counting muscle cell layers requires the identification of distinct cells, while the act of identifying vessel or wall borders for measuring can be performed based on less complex image details.

**TABLE 2:** Number of measurements acquired per specimen.

	NOX1	NOX2	HYX1	HYX2
Volume sections from LM images	17	22	14	21
Vessel segments identified in CT image	265	206	274	335
Vessel cross sections measured	179	154	170	201
Vessel cross sections analyzed for smooth muscle layer count	116	144	113	150

### 2.7 Cluster analysis

The central aspect of this study is the grouping of the vessel structures into homogeneous clusters for inter‐group comparisons. As it was uncertain which grouping method performs best for the rabbit lung, multiple established methods were employed, and their results evaluated for quality. The best‐performing method was then used for the comparisons between the animal groups.

The methods applied were generations (Ochs & Weibel, 2008) and orders (Horsfield, 1984), as well as Strahler orders (Strahler, 1957) and fractal generations (Wang & Kraman, 2004). All of these methods define the groups based on the branching pattern of each vessel tree.

The fractal generations were attributed using the Spatial Graph Extractor (https://github.com/phcerdan/SGEXT). The other metrics reported here were generated by using the Generation Analysis Toolkit (https://github.com/labode/genana_py).

In addition to these position‐based methods, a morphometry based method, namely the Gaussian mixture model (GMM; see e.g., Fraley & Raftery, 2007) in conjunction with the Bayesian information criterion (BIC; see Schwarz, 1978) was employed. Both BIC and GMM were used in their implementation in the R (R Core Team, 2018) package mclust (Scrucca et al., 2016, v5.4.7). Here, the collected lumen and wall thickness measurements were used as an input for this method. The combination of BIC and GMM is then used to find Gaussian distribution patterns in the dataset and assigns all value combinations within one such distribution to one cluster (see Figure 1c). We calculated the data of this once for each individual vessel tree, as well as for all datasets combined.

A more detailed description of all these methods can be found in Labode et al. (2022). To evaluate the grouping quality, the Davies–Bouldin index (Davies & Bouldin, 1979) in its R implementation (Walesiak & Dudek, 2020, v0.49.2) was employed.

Based on the collected measurements and graph locations of the vessels if applicable, different classifications of the datasets were calculated. Table 3 lists the different grouping methods per sample, rated by the Davies–Bouldin index. The first five classifications were calculated on each sample separately, and a global average score over all four animals was determined. Additionally, two GMMs were calculated over the combined dataset of all samples, one with four clusters and one with three. For these, only the global value is thus available. The reason for the use of two GMMs with different cluster counts is, that the BIC for a global analysis scored a distribution into four clusters slightly better than one into three clusters (−12051.69 for four and −12058.91 for three), despite all individual sample analyses identify three clusters as preferable. Thus, the Davies–Bouldin index was calculated for both cluster counts.

**TABLE 3:** Davies–Bouldin index results per grouping algorithm.

Algorithm	Groups	NOX1	NOX2	HYX1	HYX2	Global
Generations	25–32	27.46	33.48	35.61	18.78	28.83
Orders	26–32	5.82	2.72	5.89	6.29	5.18
Fractal generations	4–6	2.61	1.75	2.42	2.20	2.25
Strahler orders	4–5	1.62	1.78	1.73	2.96	2.02
GMM local	3	1.04	0.96	0.93	1.43	1.09
GMM global	4					1.36
GMM global	3					1.01

### 2.8 Statistical analysis

For intra‐ and inter‐group comparisons of the acquired measurements, both globally and between corresponding clusters for each group, the Mann–Whitney *U* test (Mann & Whitney, 1947) in its R implementation was used. This test does not require equal sample sizes and is thus applicable to the dataset. The significance level was set to 5% (*p* < 0.05). The data were used as collected. No outliers or other values were removed or changed. The individual measurements are displayed in Figures 4, 5, 6 as a scatter plot overlay to the box plots. The plots were generated using ggplot2 (Wickham, 2016).

Using the formula

(1) $$r=\frac{z}{\sqrt{N}}$$

(Rosenthal & Rubin, 1991), the effect strength was estimated in R. *z* was calculated using the R function qnorm on the *p* result of the Mann–Whitney *U* test divided by two. *N* represents the number of observations.

The effect strength serves as a measure to categorize the value shift between two significantly different distributions. Results can be attributed to one of the following categories: 0.1–0.3: weak effect, 0.3–0.5: medium effect, >0.5: strong effect (Cohen, 1992).

## 3 RESULTS

### 3.1 Clustering

The different clustering methods yielded the results displayed in Table 3. The group column lists the number of resulting clusters for each calculation. A range is given when the number changes between samples. The Davies–Bouldin index is listed both for each individual sample and as an average value in the global column. For the GMM calculations on all samples combined, this is the only result achieved.

The Davies–Bouldin index measures a global similarity between the clusters a dataset is divided into. As the goal of clustering is to create distinct clusters with as little overlap as possible, a low Davies–Bouldin score is preferable.

**FIGURE 3:**
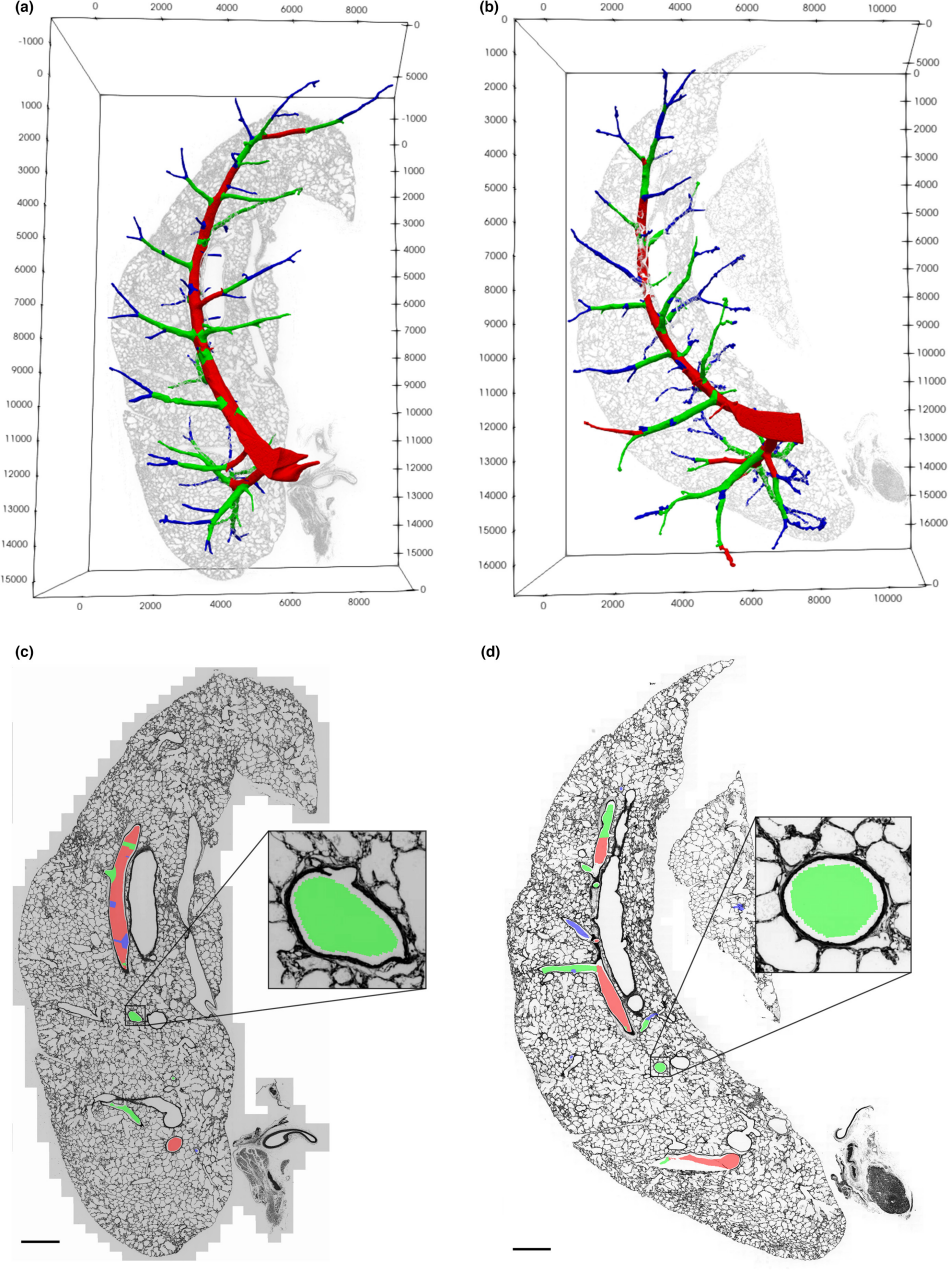
(a, b) 3D rendering of the GMM marked arterial segmentation of the lungs NOX2 and HYX2, respectively. One LM sample section is placed at the appropriate location in the volume image. (c, d) 2D rendering of the LM sections shown in (a) and (b). GMM marked segmentation information has been overlaid. Insets show exemplary vessel segments of equal cluster attribution. This combination of imaging technologies and morphological classification allows the comparison between organs. One vessel in the lower half of the slice depicted in (d) has been damaged in the cutting process. Scale in (a) and (b) in μm, scale bar (c) and (d) = 1 mm, insets are 8× magnified.

According to these results, the global GMM classification with three clusters performed best and was thus used for the following statistical analysis. The GMM results are shown in Figure 1c. The three identified clusters will be denoted to as follows: cluster 1, colored red, will be referred to as “large vessels”; cluster 2, colored green, will be referred to as “medium vessels”; cluster 3, colored blue, will be referred to as “small vessels.” Figure 3 shows the clustering results in context of the lung structure.

### 3.2 Statistical analysis

#### 3.2.1 Arterial lumen

**FIGURE 4:**
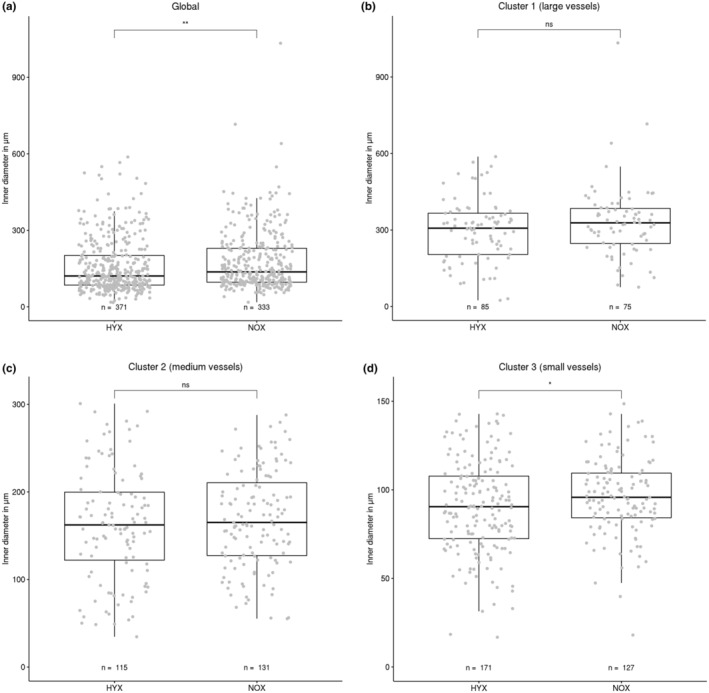
Lumen diameter comparison between HYX and NOX animal groups. Both global comparison and comparisons between clusters are displayed. Each data point represents one vessel segment. The box marks the range from the first quartile to the third quartile, the median is indicated by a horizontal line. The length of the whiskers is set to 1.5 times the interquartile range. Statistically significant differences between HYX and NOX groups are indicated by *p < 0.05 and **p < 0.01. A significant difference is both found globally and between HYX and NOX in cluster 3.

The distribution of the lumen diameter measurements between HYX and NOX group (consisting of two animals each) were compared using the Mann–Whitney *U* test. Significant differences between the groups were identified, with the NOX group exhibiting a significantly larger lumen diameter (see Figure 4).

In a global comparison of all values (Figure 4a), this is clearly evident with a *p* < 0.005. The effect strength is weak, with a value of 0.11. Thus, while a significant shift is present, the difference in value is small.

In a local comparison, in which the data points of each group were further divided into the clusters identified by the GMM, the clusters of large and medium vessels (Figure 4b,c) did not exhibit a significant difference in mean values. Only in cluster 3 (Figure 4d), consisting of the smallest vessel segments, a significant difference (*p* < 0.05) could be established. The effect strength of this shift is again weak, with a value of 0.12.

These results are in accordance with for example, Tomashefski et al. (1984) who described the decrease of the arterial diameter in BPD.

In a per animal comparison, a significant difference in the global spread of the lumen diameter between the two animals of the HYX group was noticed, while the NOX group did not exhibit such fluctuations. Considering the low number of samples per group, such a data spread is not surprising and is likely caused by different reactions of the animals to the hyperoxia treatment. This underlines the fact, that the present study is not expected to return robust data about pathological changes in BPD, but to evaluate the grouping methods use for comparison purposes.

### 3.3 Arterial wall dimensions

**FIGURE 5:**
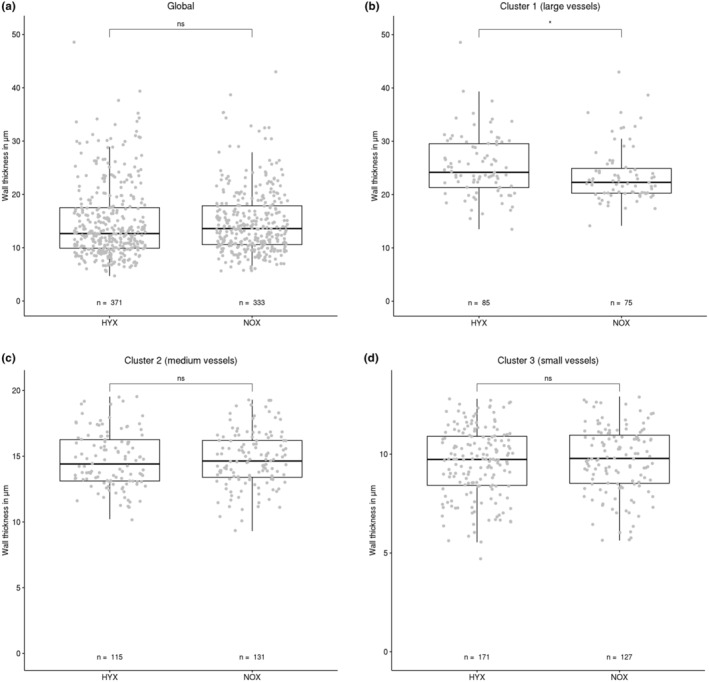
Wall dimensions of HYX and NOX animal groups. Both global comparison and comparisons between clusters are displayed. Each data point represents one vessel segment. The box marks the range from the first quartile to the third quartile, the median is indicated by a horizontal line. The length of the whiskers is set to 1.5 times the interquartile range. Statistically significant differences between HYX and NOX groups are indicated by *p < 0.05. While the global distribution is almost identical between groups, a significant difference is present between HYX and NOX in cluster 1.

Here, the approach used for the lumen diameter was replicated. The measurements collected of the arterial wall thickness were again divided into HYX and NOX groups and both globally and on a per cluster basis compared. The results can be seen in Figure 5. No significant global difference could be identified between the two groups (Figure 5a).

In the cluster‐based comparison on the other hand, significant differences between the animal groups could be measured. In cluster 1 (Figure 5b) the hyperoxia treated animals show an increased wall thickness compared with the normoxia animals. This shift is significant with *p* < 0.05 and an effect strength of 0.18. In the smaller vessels (Figure 5c,d) no significant difference in the distributions could be determined.

**FIGURE 6:**
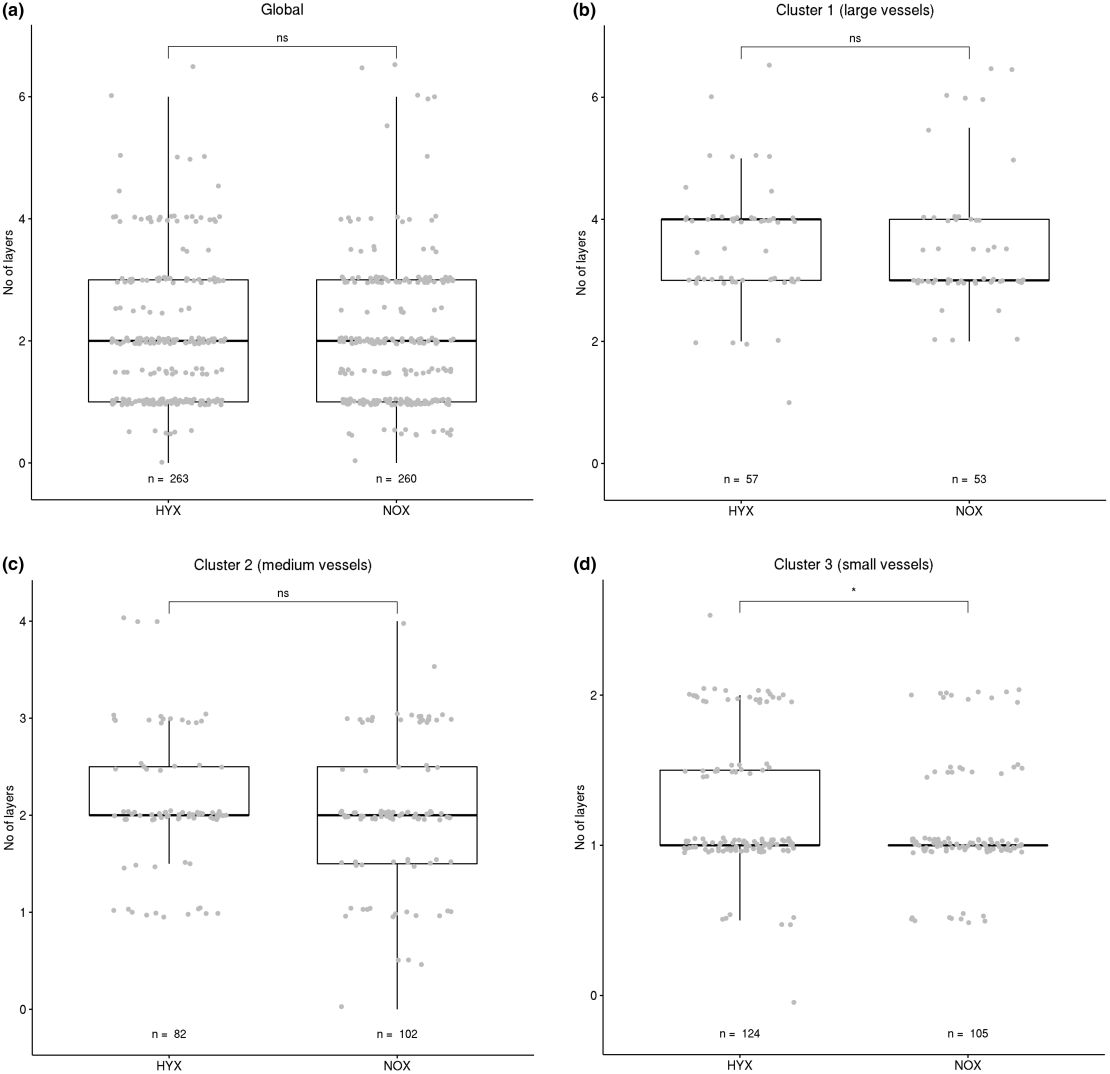
Wall layer comparison between HYX and NOX animal groups. Both global comparison and comparisons between clusters are displayed. Each data point represents one vessel segment. The box marks the range from the first quartile to the third quartile, the median is indicated by a horizontal line. The length of the whiskers is set to 1.5 times the interquartile range. Statistically significant differences between HYX and NOX groups are indicated by *p < 0.05. While the global distribution is almost identical between groups, a significant difference is present between HYX and NOX in cluster 3.

### 3.4 Arterial wall composition

To further characterize the arterial wall, the number of smooth muscle cell layers per vessel segment was counted. The distribution of the results is displayed in Figure 6. Again, a grouping into HYX and NOX datasets was conducted. Just as with the wall dimension, there is no significant global difference between both groups (Figure 6a).

On the cluster level, a difference between the small vessel clusters of the two groups could be observed (Figure 6d). In contrast to the situation in the wall thickness analysis, the HYX group exhibits a wider spread in value with an upward shift compared with the NOX group. The shift is significant with *p* < 0.05, the effect strength is weak with a value of 0.12. The larger vessel groups (Figure 6b,c) exhibit no significant changes.

## 4 DISCUSSION

### 4.1 Approach

Here, a discussion of the GMMs suitability for the study goal of finding differences between equal vessel sections is necessary. It has to be acknowledged, that grouping the vessel segments according to their morphology (instead of their location) might mask the changes that are to be identified. However, the GMM groups the vasculature, ranging from the pulmonary artery down to the precapillary vessels, into only three distinct groups. Thus, the differences between the majority of one group and its neighbor are expected to be large enough that there is no significant risk for pathological changes to lead to a misattribution of a significant number of vessels into a different group. This is evident in the provided measurements and can also be verified by comparing 3D renderings of the color‐coded vessel trees between the NOX and HYX groups.

### 4.2 Method performance

In the analysis of the lumen diameter, our method performed well. The global group comparison showed a significant difference in lumen diameter between NOX and HYX groups, with the HYX group exhibiting a lower lumen diameter overall. The presented cluster‐based analysis is able to pinpoint the significant differences to cluster 3 (the smallest vessels).

The real strength of this analysis method became visible in the wall analysis (Figures 5 and 6). While a global analysis was unable to identify any significant differences between the animal groups, a comparison of the individual clusters identified a significant difference in layer thickness in cluster 1 (largest vessels), with the NOX group exhibiting vessels with less thickness of the walls. In the wall layer analysis, the small vessels showed a significant increase in cell layer count in cluster 3 (the smallest vessels). Both of these potential local pathological morphometric changes would have been overlooked in a purely global analysis.

### 4.3 Possible usage scenarios

In the present study, the parameters used for grouping were chosen, as they change significantly on the arterial pathway through the organ. They were thus judged as representative parameters for classification of the dataset into homogeneous subgroups. They are also known to be influenced by BPD, as the referenced literature in the analysis sections discusses.

The method demonstrated in this study is however not limited to these parameters or to morphological parameters only. Other numerical properties of the structures to be grouped could be used as well, for example, the cell composition of an airway could be used for its classification. The uniformity of grouping parameters and parameters to be analyzed for pathological changes, as presented in the current study, is not a necessity either.

### 4.4 Influences of the imaging procedure

While the resolution of the μCT scans in general was good enough to identify precapillary arteries, the varying levels of image noise in the scans limited the achievable precision. Future studies should employ better quality CT scans that are becoming more and more available due to technical improvements in CT machines. A more consistent image quality would allow a more precise characterization of the overall branching structure of the vessel trees.

The shortcomings of the imaging and thus subsequently the segmentation process made an analysis challenging, especially as it resulted in large (long) vessels that were missing their smaller daughter branches, as these could not be segmented. Thus, there was less branching in certain locations of the vessel trees and, as the branching‐off points are the borders of each vessel segment, some exceptionally long vessel sections are identified as single segments. The result were major disturbances in the branching‐based categorization methods, for example, the Strahler order, as vessels were sorted into order one, while only their tip would belong there. Additionally, reduced branching inhibits the order increase and thus biases the clustering results.

The GMM was not affected by the number of nodes (so‐called branching points) and their order in the graph, but here as well, the results were limited by the data resolution. The larger (longer) segments often incorporated multiple heterogeneous subareas and thus the sample measurements taken of them became potentially less representative for their characterization.

## 5 CONCLUSION

The method demonstrated in this study was able to identify local changes that would have been undetected in a global morphometric analysis. It further allowed to locate the area of the strongest effect of global differences between HYX and NOX animals. A reduced lumen diameter was present in HYX animals. An increased wall thickness and wall layer count could be identified in different areas in the HYX animals compared with the NOX group. Both of those changes would not have been identifiable without the cluster‐based comparison approach presented in this study.

The presented method was limited by the fluctuations in μCT image quality. Future studies will need to improve this factor to achieve a higher method precision and gain a deeper insight into the pathological changes in precapillary and capillary vessels. Novel imaging technologies, such as nano‐CT and synchrotron‐CT put such improvements within reach of future projects.

In future studies, the described methods should be used to perform an evaluation on a representative sample size. This could then present a more detailed understanding of lung pathologies compared to previous, global analysis methods. As the methods presented here do not require any special preparation of the samples, they could be employed to existing sample material to extend the information gained from previous studies.

## AUTHOR CONTRIBUTIONS

JL contributed to conceptualization, methodology, formal analysis, software, validation, visualization, writing—original draft. DH, RH, YR, AG, TS, and JT contributed to resources, investigation, writing—review and editing. CM contributed to supervision, conceptualization, funding acquisition, validation, writing—review and Editing.

## FUNDING INFORMATION

The study was funded by the DFG (3118/8–1) and the German Center for Lung Research. This study was in part supported by The Research Foundation—Flanders (FWO Flanders) (grant G0C4419N) and KU Leuven (C2 grant: C24/18/101).

## CONFLICT OF INTEREST STATEMENT

The authors declare that they have no competing interests.

## CODE AVAILABILITY

Makefiles and R‐scripts are available upon request.

## ETHICS STATEMENT

All animal experiments comply with the EU Directive 2010/63/EU on the protection of animals used for scientific purposes. This study was approved by the Ethics committee for Animal Experimentation of KU Leuven, Belgium, (P081/2017).

## Data Availability

Image data are available upon request.
